# Polyarteritis nodosa with abdominal bleeding: imaging with PET/CT and angiography on the same day

**DOI:** 10.1093/rap/rkae095

**Published:** 2024-08-06

**Authors:** Kirsi Taimen, Ilpo Koskivirta, Laura Pirilä, Heikki Mäkisalo, Marko Seppänen, Topias Allonen

**Affiliations:** Division of Medicine, Centre for Rheumatology and Clinical Immunology, University of Turku and Turku University Hospital, Turku, Finland; Division of Medicine, Centre for Rheumatology and Clinical Immunology, University of Turku and Turku University Hospital, Turku, Finland; Division of Medicine, Centre for Rheumatology and Clinical Immunology, University of Turku and Turku University Hospital, Turku, Finland; Department of Gastroenterology, Turku University Hospital, Turku, Finland; Department of Clinical Physiology, Nuclear Medicine and PET, Turku University Hospital, Turku, Finland; Department of Radiology, Turku University Hospital and University of Turku, Turku, Finland

A 59-year-old male with seropositive rheumatoid arthritis, hypertension and anticoagulation due to past pulmonary embolism was hospitalized for fever (39°C) and myalgia. His CRP level was 190 mg/l and he had haematuria and proteinuria, but infection screening and ANCA remained negative. One week after admission he experienced a sudden life-threatening abdominal catastrophe. ^18^F fluorodeoxyglucose (FDG)-PET/CT was performed earlier on the same day, before the abdominal symptoms appeared. PET/CT detected increased FDG uptake in the peri- and intramuscular arterial tree of the lower extremities, indicating vasculitis in middle-sized arteries, as well as inhomogeneous uptake in the liver (Fig. 1A). During the abdominal crisis, the CT scan showed haematoma and active bleeding of the right liver lobe (Fig. 1B). Digital subtraction angiography revealed multiple hepatic artery microaneurysms and stenoses (Fig. 1C). These findings were consistent with polyarteritis nodosa (PAN). He underwent successful emergency hepatic arterial embolization and was later treated with a molecular adsorbent recirculating system (MARS) for temporary liver failure. Immunosuppression was started with methylprednisolone and cyclophosphamide. Hepatitis serology was negative. Subsequent genetic testing for *UBA1* and *ADA2* was normal.

**Figure 1.**
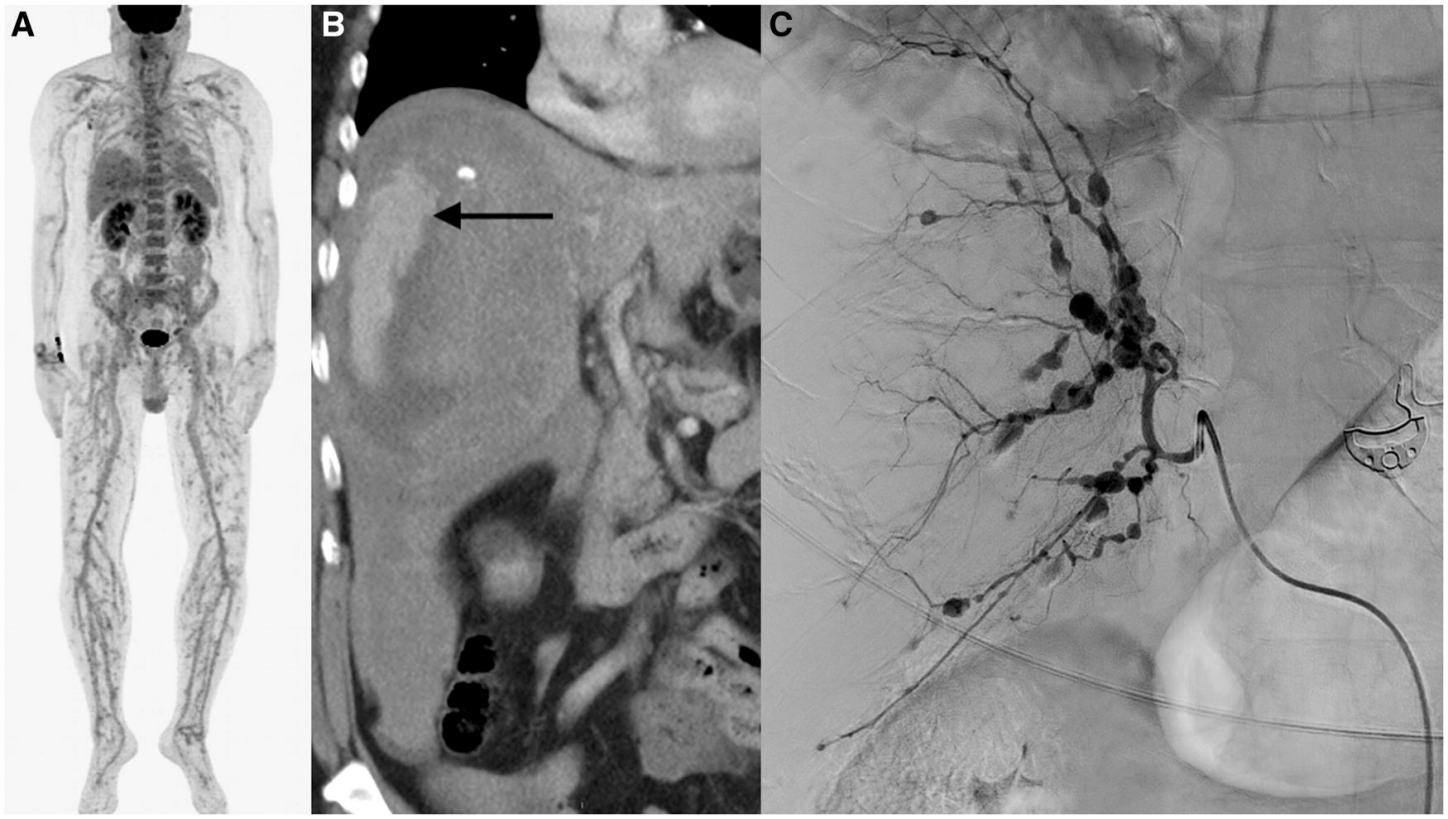
(**A**) PET/CT. The maximum-intensity projection shows increased FDG uptake in the peri- and intramuscular arterial tree of the lower extremities and inhomogeneous uptake in the liver. (**B**) CT of the abdomen, coronal view. Haematoma in the right lobe of the liver (arrow). (**C**) Angiography of the hepatic artery showing microaneurysms and stenoses

In PET/CT, the linear hypermetabolic FDG uptake in the lower extremities resembles findings previously described in a small PAN patient series, confirming the importance of this observation [1].

## Data Availability

All the relevant data to the clinical image have been shared. Additional information is available on reasonable request from the corresponding author.
